# Relationship Between Thoracic Spine Rotation Range, Trunk Contralateral Flexion Angle, and Maximum Elbow Valgus Torque During Pitching

**DOI:** 10.7759/cureus.80059

**Published:** 2025-03-04

**Authors:** Shun Okamura, Kai Iida

**Affiliations:** 1 Department of Physical Therapy, Faculty of Health Science Technology, Bunkyo Gakuin University, Saitama, JPN

**Keywords:** baseball, elbow valgus torque, lumbar rock rotation, range of motion (rom), trunk contralateral flexion angle

## Abstract

Introduction: Excessive trunk contralateral flexion increases elbow valgus torque during pitching. However, the cause of this excessive contralateral flexion is unclear. This study aimed to clarify the relationship between the lumbar rock rotation test (LLR-t), trunk contralateral flexion angle during shoulder joint in maximum external rotation, and maximum elbow valgus torque. Methods: This descriptive laboratory study included 21 healthy male baseball players with pitcher experience in the LLR-t. Angles were measured using an inclinometer. The maximum elbow valgus torque during pitching was measured using an inertial measuring unit (IMU), and the trunk contralateral flexion angle during shoulder joint in maximum external rotation (MER) was measured using ImageJ (National Institutes of Health, Bethesda, Maryland, United States) after video recording. Results: The throwing side LLR-t showed a significant negative correlation (r=-0.51, p=0.02) with the trunk contralateral flexion angle during MER and a significant negative correlation (r=-0.45, p=0.04) with the maximum elbow valgus torque during throwing. MER trunk contralateral flexion angle showed a significant positive correlation (r=0.47, p=0.03) with maximum elbow valgus torque during pitching. Conclusions: The LLR-t may be a useful method of measuring thoracic spine rotation range of motion in athletes with increased trunk contralateral flexion angle and high maximal elbow valgus torque during pitching.

## Introduction

The throwing motion involves transferring the energy generated by the lower limbs to the trunk and upper limbs. Fleisig et al. reported a shoulder joint internal rotation torque of 67 ± 11 Nm and an elbow varus torque of 64 ± 12 Nm during pitching [1]. Excessive and repeated application of such forces during pitching is considered to lead to injury. Regarding the relationship between elbow joint disorders, which occur frequently in baseball, and elbow joint valgus torque (EVT) [2], EVT was found greater in players with elbow joint disorders. A three-dimensional (3-D) motion analyzer is commonly used to measure the load on upper extremity joints and can only be used in specific environments. Regarding EVT during pitching, Aguinaldo et al. reported that it is related to the power of the trunk and upper limb and the onset time of trunk rotation movement [3]. Okamura et al. reported that a larger throwing-side LLR-t angle is associated with a lower normalized maximum EVT (nMEVT) [4]. Therefore, it is important to consider not only the injury site but also the influence of the trunk in physical therapy.

Previous reports showed that pitching velocity and elbow varus torque increased with increasing trunk contralateral flexion angle to the body side in college and high school baseball players, indicating that greater trunk lateral flexion during pitching enhances pitch velocity but also increases EVT, potentially elevating the risk of elbow joint disorders [5,6].

In clinical practice, athletes with increased contralateral trunk lateral flexion angles often have low values for the lumbar rock rotation test (LLR-t), which measures thoracic spine rotation range of motion. The LLR-t measurement position assesses the thoracic rotation angle with the upper limb elevated. This position closely approximates the throwing motion and is considered useful for baseball players. However, the relationship between LLR-t and throwing motion has not been fully elucidated. In a previous study on the relationship between trunk contralateral flexion angle during pitching and trunk function, Myrick et al. reported contributing factors to increased trunk lateral flexion during pitching were not associated with trunk flexibility or muscle strength [7]. In contrast, Oyama et al. reported a relative decrease in oblique muscle strength on the throwing side [8]. Thus, no definitive conclusions have been reached regarding the relationship between trunk function and pitching motion. However, since the LLR-t angle is associated with elbow valgus torque, it may also be related to trunk contralateral flexion angle during pitching.

The primary objective of this study was to clarify the relationship between the LLR-t angle and trunk contralateral flexion angle during pitching and nMEVT. Additionally, the secondary objective was to evaluate whether LLR-t measurement is useful for assessing the risk factors for elbow injuries and improving pitching performance. We hypothesized that both the contralateral trunk lateral flexion angle during pitching and EVT increase in athletes with low valgus of the throwing LLR-t angle.

## Materials and methods

This was a descriptive laboratory study conducted at Tokai University Shizuoka Shoyo High School, Shizuoka, Japan, and Saitama Prefectural Fujimino High School, Fujimino, Japan, from December 2023 to January 2024. The study was approved by the Health Sciences Research Ethics Committee of Bunkyo Gakuin University (approval number: 2022-0012). The participants were provided with an explanation of the research objectives and measurements were taken after obtaining their consent.

Participants

A total of 21 healthy male high school baseball players (age: 16.7 ± 0.5 years, height 172.6 ± 4.8 cm, weight 69.1 ± 6.3 kg, baseball history 9.2 ± 1.9 years) who were able to cooperate were enrolled in the study. All participants were members of their high-school baseball team. The exclusion criteria were (i) pain in the shoulder and elbow joints within the past year, which made pitching difficult, and (ii) players with an underhand or sidearm pitching motion. Players who started playing baseball in high school were included. None of the participants complained of pain in the shoulder or elbow joints during pitching. The measurements in this study were conducted during the off-season (i.e., January).

Sample size

A priori power analysis was conducted to determine the appropriate sample size required to achieve statistical significance with a power of 80% (1 - β). This analysis was performed using G*Power 3.1.9.4 (http://www.gpower.hhu.de/). To examine the correlations between the LLR-t angle, trunk contralateral flexion angle during pitching, and nMEVT, the sample size was calculated using the following settings: exact effect size, 0.49; α, 0.05; power (1 - β), 0.8. The effect size was set based on previous reports [4]. A total of 30 participants were required for this study. However, as the measurements were conducted during the off-season, we were only able to recruit 21 participants who met the inclusion criteria and agreed to participate in the study.

Thoracic spine rotation range of motion

The LLR-t measurement method was based on Okamura et al.'s reports [9]. LLR-t measurements were conducted after the participants completed their usual warm-up routine, separate from pitching. Participants started on the floor, on all fours, with the buttocks touching the heels and the forearms touching the floor. Subsequently, one hand was placed on the back of the head (the measuring hand) while the head was held in the intermediate position, and the trunk was automatically rotated to the maximum angle. The height of the thoracic rotation angle was measured at the height of the first and second thoracic vertebrae, using an inclinometer (blue slant dial type; Shinwa Rules Co., Ltd. Niigata, Japan). Measurements were conducted three times alternately on each side, for a total of six measurements. The average of the three measurements on each side was used as the representative value for each participant; the rotation angle toward the dominant hand was defined as the throwing side, and the rotation angle toward the non-dominant hand was defined as the non-throwing side. The intraclass correlation coefficient (ICC) was calculated to assess the intra-examiner reliability of the LLR-T angles in this study. The ICC (1,3) was 0.97 (95% confidence interval (CI): 0.93-0.99) for the right angle of rotation and 0.97 (95%CI: 0.95-0.99) for the left angle of rotation, indicating high reliability.

Measurement of trunk contralateral flexion angle and MEVT during pitching

The trunk contralateral flexion angle and MEVT during pitching were measured in an indoor field covered with artificial turf. The participants wore uniforms and rubber-soled shoes. A glove was worn on the non-throwing side, and a practice ball (140 g; Rawlings Sporting Goods, St. Louis, Missouri, United States) was used for pitching. Measurements of trunk contralateral flexion angle during pitching and MEVT were conducted after the LLR-t measurement.

**Figure 1:**
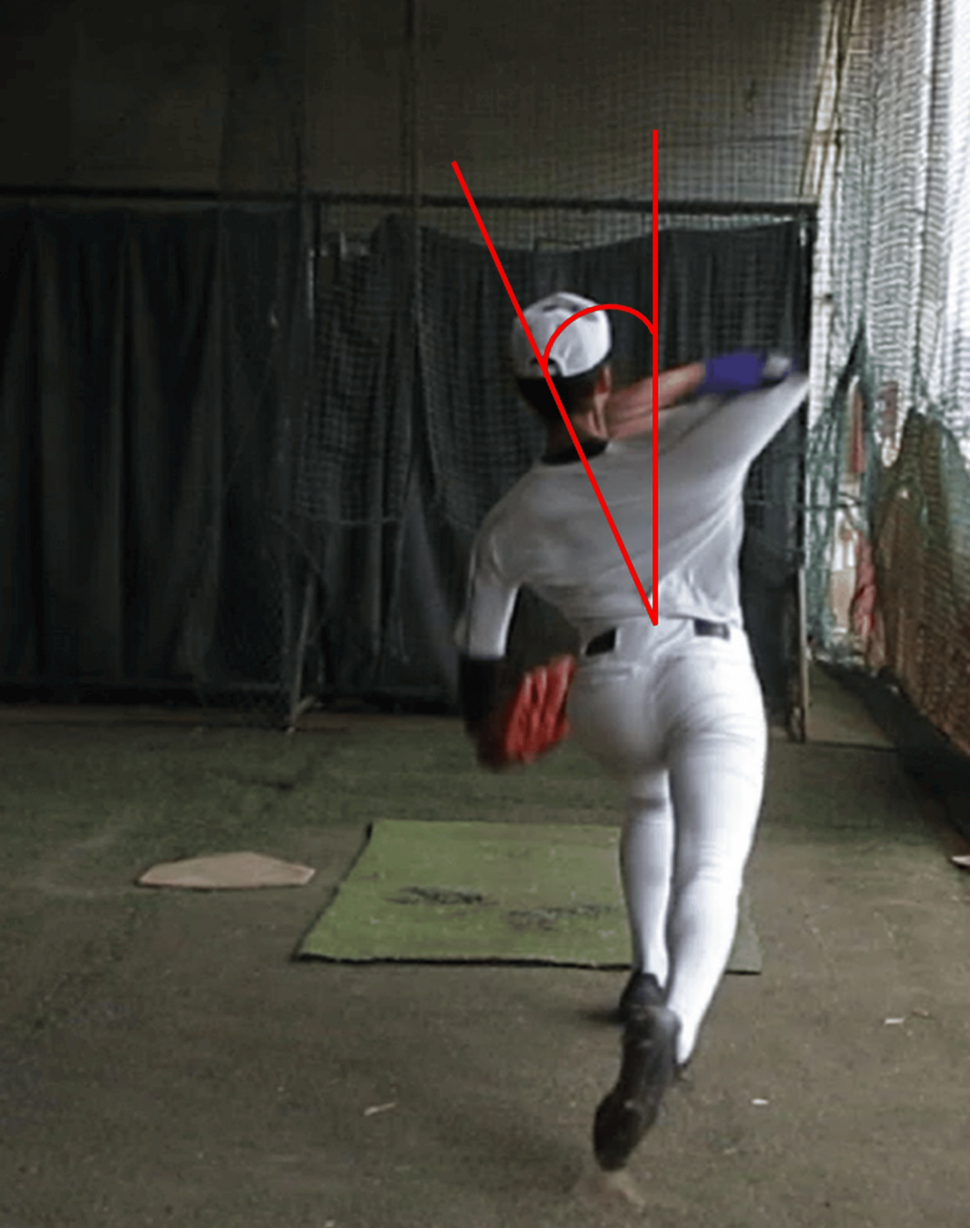
Trunk contralateral flexion angle during shoulder joint in MER MER: maximum external rotation

Before the measurements, participants performed their usual warm-up catch routine, which was limited to 20 minutes. Participants were instructed to perform their usual warm-up routine for 30 minutes before pitching. The measurement task was to throw five pitches with all their might toward a pitching net set 5 m away, and the average value of the three attempts for pitches 2-4 was analyzed. The interval between pitches was set at 20 seconds. Participants were instructed to “pitch with all your might.” The contralateral trunk flexion angle during shoulder joint in maximum external rotation (MER) was measured using a digital camera (30 frames per second (fps), Canon IXY 650; Canon Inc., Tokyo, Japan) placed 3 m behind the pitcher's plate to capture a video of the pitch motion. Subsequently, the frame that became MER on the video was used to calculate the trunk contralateral flexion angle during MER using image analysis software (ImageJ; National Institutes of Health, Bethesda, Maryland, United States) (Figure 1). The trunk contralateral flexion angle was calculated by drawing a line vertically from the floor and intersecting the vertical line of the trunk, defining the contralateral side as positive and the throwing side as negative. In this study, the ICC was calculated to assess the intra- and inter-examiner reliability of the trunk contralateral flexion angle during MER. The results showed that the ICC (1,2) was 0.97 (95%CI: 0.94-0.99) and the ICC (2,1) was 0.98 (95%CI: 0.95-0.99) and the measured items also showed high reliability.

**Figure 2:**
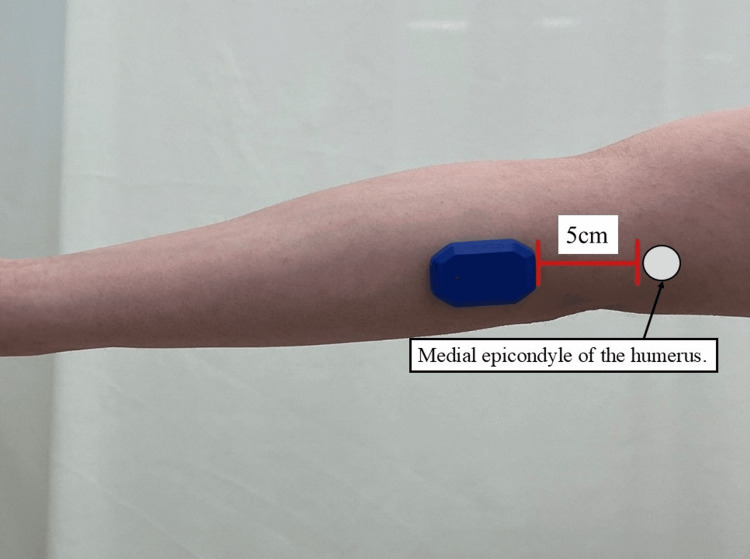
The IMU was attached 5 cm distal to the medial epicondyle of the humerus IMU: inertial measurement unit

Next, MEVT was measured using an inertial measurement unit (IMU) (PULSE Throw Workload Monitor; Driveline Baseball, Kent, Washington, United States) (Figure 2). This sensor is a detachable inertial measurement device with a triaxial accelerometer and gyroscope recorded at 1000 Hz. Similar to the report by McHugh et al. [10], the IMU was attached 5 cm distal to the medial epicondyle of the humerus. The measurement accuracy when repeatedly throwing fastballs using this device has been reported as between 96.9 [11] and 100% [12]. Additionally, studies comparing absolute elbow joint torque values obtained from IMU and motion capture have reported that IMU measurements tend to underestimate torque. Bobby et al. [13] reported an underestimation of 38.7%, while Camp et al. [14] found an underestimation ranging from 20% to 60%. To exclude the influence of body physique on the MEVT obtained using the PULSE, we referred to a previous report [3] and normalized the MEVT by multiplying it by height and weight.

Statistical analysis

Statistical analysis was performed using IBM SPSS Statistics for Windows, Version 25.0 (2017; IBM Corp, Armonk, New York, United States) to examine the relationship between the LLR-t angle and the trunk contralateral flexion angle and MEVT during MER. Referring to the report by Albeiero et al. [15], the degree of correlation was defined as weak (0.1 < r < 0.3), moderate (0.3 < r < 0.5), and strong (r > 0.5). The statistical significance level was set according to the risk rate, being < 5%.

## Results

The mean values of the participants’ LLR-t angle, trunk contralateral flexion angle during the MER, and MEVT are listed in Table 1. The LLR-t was 55.0 ± 10.1° on the throwing side and 53.7 ± 10.7° on the non-throwing side. At MER, the trunk contralateral flexion angle was 22.1 ± 7.9°, and the MEVT was 0.34 ± 0.06 (Nm/kg*m).

**Table 1:** Average value of each measured parameter LLR-t: lumbar rock rotation test; MER: maximum external rotation

Measurement parameter	Mean (SD)
LLR-t throwing side（degree)	55±10.1
LLR-t non-throwing side（degree)	53.7±10.7
MER trunk lateral flexion angle（degree)	22.1±7.9
Maximum elbow valgus torque（Nm/kg*m）	0.34±0.06

The correlation between the angles of LLR-t and trunk contralateral flexion during MER and the maximum elbow valgus torque are presented in Table 2. We found a strongly significant negative correlation (r=-0.51, p=0.02) between the LLR-t angle and trunk contralateral flexion angle at MER with the throwing side of the LLR-t. A moderately significant negative correlation (r=-0.45, p=0.04) was found between the LLR-t throwing side and the MEVT during pitching. Finally, a moderately significant positive correlation (r=0.47, p=0.03) was found between the MER trunk contralateral flexion angle and MEVT during pitching. No other correlations were observed.

**Table 2:** Correlations between each measurement parameter *Indicates p<0.05,**Indicates ｐ<0.01 LLR-t: lumbar rock rotation test; MER: maximum external rotation

Measurement parameter	LLR-t throwing side（degree)	LLR-t non-throwing side（degree）	MER trunk lateral flexion angle（degree）	Maximum elbow valgus torque（Nm/kg*m）
LLR-t throwing side (degree)	1	0.83**	-0.51*	-0.45*
LLR-t non-throwing side (degree)	-	1	-0.24	-0.31
MER trunk lateral flexion angle (degree)	-	-	1	0.47*
Maximum elbow valgus torque (Nm/kg*m）	-	-	-	1

## Discussion

This study evaluated the relationship between LLR-t, which measures the rotation angle of the thoracic spine, contralateral trunk flexion angle during MER, and MEVT. The results revealed that in athletes with a low LLR-t throwing side angle, the angle of trunk flexion to the contralateral side during MER and the maximum elbow valgus torque were increased.

Myrick et al. found no relationship between the angle of trunk contralateral flexion to the side of the body and that of trunk rotation during pitching [7]; however, we believe that the relationship found in the present study was due to differences in the upper limb position during measurement. Myrick et al. calculated the angle of trunk rotation in the sitting position with their arms crossed in front of their chest [7]. Contrastingly, LLR-t measured the angle of thoracic spine rotation on all fours with the upper limbs raised; the results were determined by the ipsilateral pectoralis major and minor muscles, which limited thoracic spine rotation and were extended in this position. There was a negative correlation between the throwing-side LLR-t angle, trunk contralateral flexion angle at the MER, and MEVT. The increase in trunk lateral flexion angle during MER in athletes with a lower throwing-side LLR-t angle may result from a reduced relative torsion angle between the trunk and pelvis ("trunk-pelvis separation") at stride foot contact (SFC). 

Lin et al. reported that the pelvic and trunk torsion angles were smaller in athletes who showed early rotational motion to the nonthrowing side during pitching, suggesting that a decrease in the relative pelvic and trunk torsion angles may result in early trunk rotational motion to the nonthrowing side during SFC [16]. Another study reported that compared to the control group, the group exhibiting early trunk rotation toward the non-throwing side had a greater shoulder abduction angle at SFC and a smaller angle at MER [17]. Based on these findings, the increased trunk lateral flexion angle toward the non-throwing side in players with a lower throwing-side LLR-t angle is considered a compensatory mechanism for the reduction in shoulder abduction angle at MER caused by early trunk rotation toward the non-throwing side. However, pitchers with a greater shoulder abduction range of motion due to factors such as joint laxity may be less likely to exhibit compensatory lateral flexion. Additionally, individual differences in hip and trunk flexibility and muscular strength may influence changes in trunk-pelvis separation, potentially leading to variations in the presence and extent of compensatory motion. An increased trunk contralateral flexion angle increased the MEVT, similar to what was shown in previous reports. [5.6]

Additionally, Aguinaldo et al. [18] and Sabick et al. [19] have reported that EVT is positively correlated with the shoulder joint external rotation angle during MER. However, these reports examined the relationship between the angle of the humerus and the trunk and did not consider the posterior scapular tilt angle relative to the thorax. Miyashita et al. reported that external rotation of the scapulohumeral joint, posterior scapular tilt, and thoracic spine extension movements were added [20]. In this study, we postulated that the LLR-t used is not a pure thoracic rotation movement but involves a slight thoracic extension movement, scapular posterior tilt, and upward rotation movement along with the former. Therefore, we postulated that the MEVT is smaller in players with higher values of the throwing-side LLR-t angle because of the greater contribution of the thoracic extension angle and scapulothoracic joint posterior tilt angle during MER, and the decrease in the scapulohumeral joint external rotation angle. The secondary objective was to examine whether LLR-t measurement is useful as an evaluation method for preventing elbow injuries in baseball. The results showed a negative correlation between the throwing-side LLR-t angle and nMEVT. Since increased EVT is associated with elbow injuries, these findings suggest that LLR-t measurement may be useful for assessing the risk of increased EVT. This result is consistent with previous reports [4].

This study has four limitations. The first limitation is that the scapular angle during MER was not measured. This factor should be included in future studies because the scapular angle and movement are important in the pitching motion. The second limitation concerns the IMU used in this study which measured the MEVT. Although the EVT is at its maximum near the time of MER, other forces are also applied to the elbow joint; therefore, the throwing motion should be observed from multiple angles. It has been previously reported that the IMU measurements of MEVT tend to underestimate values compared to motion capture [13,14]. Therefore, careful consideration is required when directly using IMU-derived measurements. Moreover, although the MEVT measured using the IMU is likely to occur near MER, the exact timing of the measurement remains unknown. Therefore, careful consideration is required when interpreting the results. Third, ball velocity, an indicator of pitching performance, was not measured. There is also an index of pitching efficiency, which is calculated by dividing the EVT by the pitching velocity [21]. The relationship between pitching velocity and pitching efficiency should be examined. Fourth, the study included only 21 high school participants. Since growth rates vary among high school athletes, future studies should include a larger sample size and examine different age groups, such as collegiate players, for further validation.

## Conclusions

The findings of this study demonstrated a significant correlation between the throwing-side LLR-t angle and both the trunk contralateral flexion angle during pitching and MEVT. Additionally, a positive correlation was observed, indicating that a greater contralateral trunk flexion angle during pitching was associated with increased EVT. These results suggest that LLR-t may be a useful tool for assessing the thoracic spine rotation range of motion in baseball pitchers, particularly those exhibiting excessive trunk contralateral flexion and high EVT. By incorporating LLR-t measurements into routine screening and training programs, coaches and medical professionals may be able to identify athletes at higher risk of elbow injuries and implement targeted interventions to improve thoracic spine mobility and optimize pitching mechanics, potentially reducing excessive elbow valgus torque and injury risk. Future research should explore specific exercises aimed at enhancing LLR-t to further develop effective training strategies.
